# Supplementary figures and images for: Coronatine inhibits stomatal closure and delays hypersensitive response cell death induced by nonhost bacterial pathogens
Source: PeerJ. 2013 Feb 12;1:e34. doi: 10.7717/peerj.34 (PMC3628748; doi:10.7717/peerj.34)

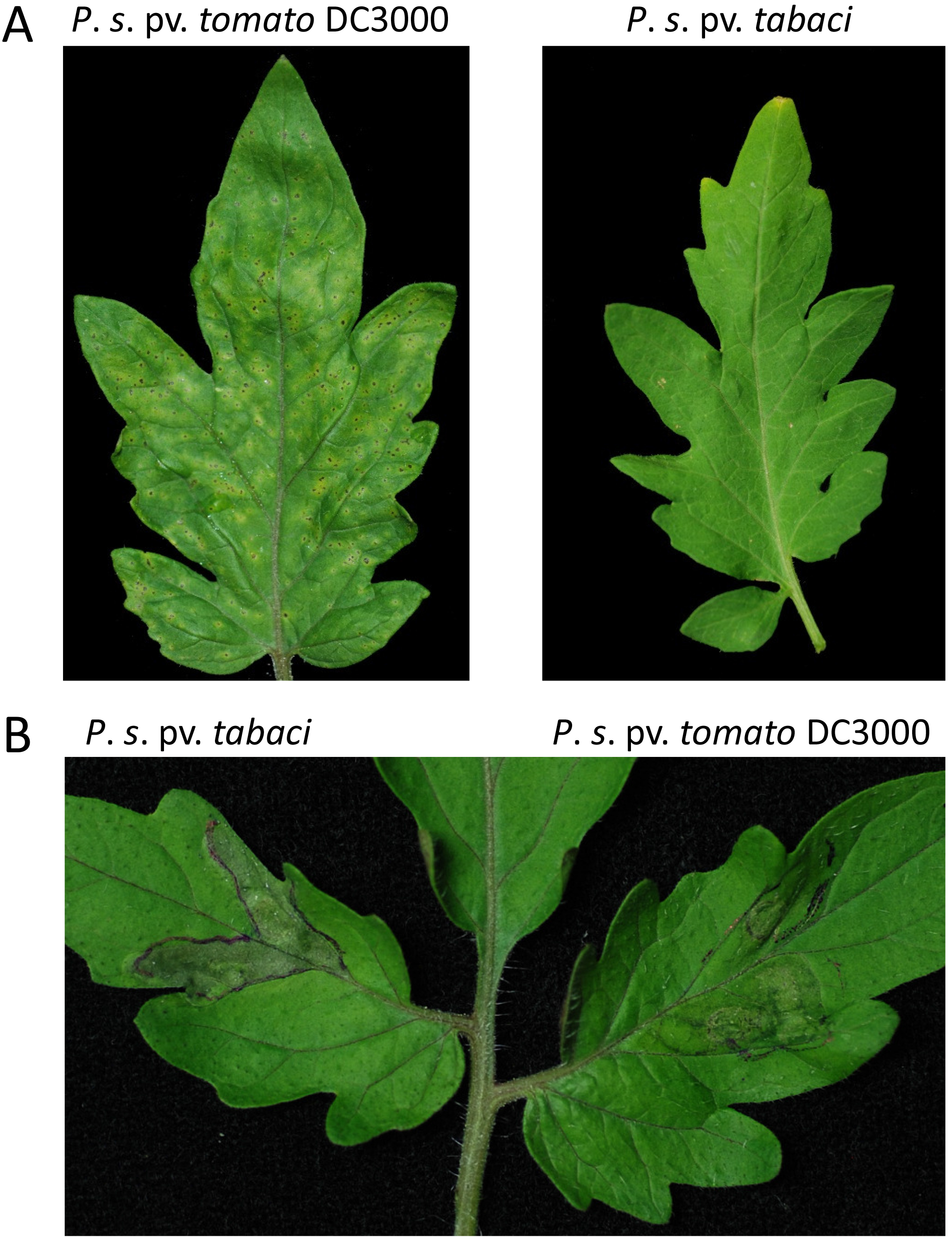

Supplement: Supplemental Figure S1 — Development of disease symptoms by host pathogen P. syringae pv. tomato DC3000 in tomato. (A) Tomato leaves were spray-inoculated with P. syringae pv. tomato DC3000 (5 × 10^3^ CFU/ml) and P. syringae pv. tabaci (5 × 10^3^ CFU/ml), and imaged five days after inoculation. (B) The nonhost HR cell death by P. syringae pv. tabaci in tomato. Tomato leaves were syringe-infiltrated with host pathogen P. syringae pv. tomato DC3000 (6 × 10^2^ CFU/ml) or nonhost pathogen P. syringae pv. tabaci (6 × 10^2^ CFU/ml). The nonhost HR cell death was observed 48 h after infiltration. [file peerj-01-34-s001.jpg]
