# Supplementary material for: Coronatine inhibits stomatal closure and delays hypersensitive response cell death induced by nonhost bacterial pathogens
Source: PeerJ. 2013 Feb 12;1:e34. doi: 10.7717/peerj.34 (PMC3628748; doi:10.7717/peerj.34)
Supplement: Supplemental Table 1 [file peerj-01-34-s002.docx]

Supplemental Table 1. List of primers and corresponding genes used for expression analysis

| **Accession #** | **Sequence Definition** | **FW primer** | **RV primer** |
| --- | --- | --- | --- |
| M69247 | *PR1a* : *Pathogenesis-related protein 1a* | CACAAAACTATGCCAACTCAAGAGC | GCCTCTCGGACACCCACAAT |
| M69248 | *PR1b* : *Pathogenesis-related protein 1b* | CACAAAACTATGCCAACTCAAGAGC | TCTCCCCAGCACCAGAATGAATC |
| M80608 | *PR2b* : *Pathogenesis-related protein 2b* | CGAGATGGTGGGTACAGAAGAAC | CAAGATTGGAAGTGCCAGTAACAGG |
| DQ149918 | *ICS1 : Isochorismate synthase 1* | TCGCCGGCATTCATTGGAAACA | AAAGCCCGTGCATCTTCTGT |
| U37840 | *LoxD*: *lipoxygenase 3* | GACTGGTCCAAGTTCACGATCC | ATGTGCTGCCAATATAAATGGTTCC |
| AI486721 | *OPR3*: *12-oxophytodienoic acid reductase 3* | TTGGCTTAGCAGTTGTTGAAAG | TACGTATCGTGGCTGTGTTACA |
| BE449710 | *α-tubulin 4* | AAACAGACCGGCATTTTACAG | GGTGATGATGAAGCAGATGGT |
